# Urban violence is the biggest cause of fatal work-related accidents in Brazil

**DOI:** 10.11606/S1518-8787.2017051000296

**Published:** 2017-12-04

**Authors:** Ricardo Cordeiro, Verônica Gronau Luz, Élida Azevedo Hennington, Ana Cláudia Alves Martins, Luís Fernando Tófoli

**Affiliations:** I Universidade Estadual de Campinas. Faculdade de Ciências Médicas. Departamento de Saúde Coletiva. Campinas, SP, Brasil; II Universidade Federal da Grande Dourados. Faculdade de Ciências da Saúde. Curso de Nutrição. Dourados, MS, Brasil; III Fundação Oswaldo Cruz. Escola Nacional de Saúde Pública. Centro de Estudos da Saúde do Trabalhador e Ecologia Humana. Rio de Janeiro, RJ, Brasil; IV Universidade Estadual de Campinas. Faculdade de Ciências Médicas. Programa de Pós-Graduação em Saúde Coletiva. Campinas, SP, Brasil; V Universidade Estadual de Campinas. Faculdade de Ciências Médicas. Departamento de Psicologia Médica e Psiquiatria. Campinas, SP, Brasil

**Keywords:** Accidents, Occupational, mortality, External Causes, Violence, Occupational Health, Acidentes de Trabalho, Mortalidade, Causas Externas, Violência, Saúde do Trabalhador

## Abstract

**OBJECTIVE:**

To quantify the occurrence of deaths directly associated with urban violence among fatal work-related accidents.

**METHODS:**

Verbal autopsies were performed with the relatives and coworkers of residents of Campinas, state of São Paulo, Brazil, who died from external causes in 2015. We have also analyzed police reports and reports of the Legal Medical Institute related to these deaths.

**RESULTS:**

We have identified 82 fatal work-related accidents in Campinas in 2015, of which 25 were murders, 35 were traffic accidents not directly related to work activities, and three were suicides at work. The proportional mortality rate for homicides, traffic accidents, and suicides among fatal work-related accidents was estimated at 30.5%, 42.7%, and 3.7%, respectively.

**CONCLUSIONS:**

Urban violence accounted for three-fourths of the fatal work-related accidents recorded in the period studied.

## RESUMO

**OBJETIVO:**

Este estudo visou quantificar a ocorrência de mortes diretamente associadas à violência urbana dentre os acidentes de trabalho fatais.

**MÉTODOS:**

Foram realizadas autópsias verbais com familiares e colegas de trabalho de moradores de Campinas, SP, falecidos por causas externas no ano 2015. Também foram analisados boletins de ocorrência e laudos do Instituto Médio Legal relativos a essas mortes.

**RESULTADOS:**

Foram identificados 82 acidentes de trabalho fatais em Campinas em 2015, dos quais 25 foram assassinatos, 35 foram acidentes de trânsito não diretamente decorrentes de atividades laborais e três foram suicídios no trabalho. A mortalidade proporcional por homicídios, acidentes de trânsito e suicídios entre os acidentes de trabalho fatais foi estimada em 30,5%, 42,7% e 3,7%, respectivamente.

**CONCLUSÕES:**

A violência urbana foi responsável por 3/4 dos acidentes de trabalho fatais contabilizados no período estudado.

## INTRODUCTION

Work-related accidents (WA) are the greatest health problem of Brazilian workers. Only in 2013, in its most recent consolidation, the Brazilian Ministry of Social Security registered the occurrence of 702,685 typical work-related accidents and those related to commutes, throughout the national territory^12^. Among those affected, 2,797 died as a result of the accident^12^, and most of them were young and productive workers. We highlight that these data are notoriously underreported in Brazil, as researchers have shown over the last decades^9^ ^,^ ^17^ ^,^ ^18^.

Traditionally, two important causes for this underreporting have been identified. On the one hand, there is no single, efficient system that centralizes WA information in Brazil. The different existing systems have limited effectiveness and little information exchange between them. On the other hand, the more specific database of the Ministry of Social Security ignores work-related accidents that occur not only among self-employed persons, public employees, and entrepreneurs, but also those occurring in the informal Brazilian economy, which covered 44.2 million informal workers in 2012, that is 56% of the workforce at the time^21^, and this number continues to grow proportionally.

However, two decades ago, the growth of violence in Brazil and the difficulty in identifying its reflexes on the working population have been suggested as relevant factors both for the occurrence of work-related accidents and for its underreporting in the country. Many fatal work-related accidents are not recognized as related to work, appearing in official statistics as common homicides and accidents in general.

In Brazil, deaths from external causes reached 2 million persons between 1980 and 2000, a period in which homicide mortality increased by 130%^25^. In the early 2000s, mortality from external causes declined, rising again after 2008. As it cannot be avoided, the increase in violence has an impact on the world of work, contributing significantly to the shaping of the mortality profile of Brazilian workers in recent decades. Several studies carried out in this period have shown an increasing participation of homicides, robberies, suicides, stray bullets, traffic accidents, kidnappings, and conflicts with criminals, coworkers, clients, and users as triggers of fatal work-related accidents^3^ ^,^ ^10^ ^,^ ^20^. Part of these problems is easily characterized as directly related to work, such as a security guard who dies shot while protecting the assets of the company during a robbery. At other times, the relationship is less direct, as in the case of a worker who is a kidnapping and murder victim when he or she leaves work.

Currently, the investigation of occupational mortality in Brazil remains relevant, whose severity is only touched by the official statistics available. Therefore, we also need to understand and size its relationship with violence associated with crime.

In view of the increasing violence experienced in large urban centers, the magnitude of the occurrence of work-related accidents in the country, and the relationship between these two problems, this study aimed to quantify, among the work-related accidents, the occurrence of deaths directly associated with urban violence, contributing for a better understanding of the Brazilian general situation.

## METHODS

This study was carried out in Campinas, a municipality located 96 kilometers northwest of the city of São Paulo. Its population in 2015 was estimated at 1,164,098 inhabitants^4^, being the third most populous city in the state of São Paulo and the fourteenth in the country. Its Human Development Index was 0.805 in 2010, high in the Brazilian context and one of the highest in the state. The city is a relatively developed industrial and technological pole that reflects well the uncooperative coexistence between wealth and poverty in great Brazilian cities.

The Municipal Health Department of Campinas (SMS) routinely receives, from multiple sources, all the death certificates of the deceased residents of the municipality. These certificates are reviewed, complemented, and corrected when needed in light of information obtained from hospitals, the Death Verification Service, and the Legal Medical Institute (IML) of the city, reclassifying the basic causes of death according to the rules of the 10^th^ Review of the International Classification of Diseases (ICD-10)^16^.

We received, from a partnership with the SMS, the content of all death certificates of persons aged between 15 and 65 years who lived in the municipality of Campinas and who died in any part of the national territory between January 1, 2015 and December 31, 2015, whose underlying cause of death was classified, after review, as an external cause (that is, within Chapter XX of ICD-10). Using the information on the place of residence, in part III of the death certificate, we located the family of the deceased person.

The information for this work was collected mainly from semi-structured interviews conducted by interviewers trained in verbal autopsy^1^. Predominantly, we interviewed close relatives of the deceased resident (parents, spouses, siblings, children), recording the information obtained. The questionnaire used included data on race, religion, educational level, occupation, work situation, and use of alcohol, tobacco, and illicit drugs, as well as open fields for the free narrative of the interviewees about the circumstances that led their relatives to death. Whenever necessary and possible, we similarly interviewed neighbors, friends, and coworkers of the deceased person. The number of interviews made for each identified death varied according to the need for completeness of data and the availability of informants. Together with the data obtained in the interviews, we also collected information from the analyses of necropsy reports performed by the IML of Campinas and other regions and copies of the police reports annexed to these reports, as well as information published in the written and spoken press of Campinas about the investigated deaths. Meetings with the entire research team, including field researchers, were conducted weekly in order to describe and discuss the deaths analyzed and assist in the final classification of the cases found.

We defined as formal workers those who, at the time of death, had a registered formal contract (e.g., salaried employees with a formal contract, regulated municipal, state, and federal civil workers with their own pension scheme, resident physicians, regulated autonomous professionals, such as self-employed professionals, entrepreneurs, and business associates, formally registered and with social security). An informal worker was defined as anyone who worked without a formal employment contract in the 30 days before death.

Work-related accidents were defined, according to the Department of Health Surveillance of the Ministry of Health (2014), as “those that happen in the exercise of the work activity, or in the commute from home to work”^13^ (p.752). Fatal work-related accidents were defined as “those that lead to death immediately after its occurrence or that occur later, at any time, in a hospital environment or not, provided that the basic, intermediate, or immediate cause of death was due to the accident”^13^ (Ministério da Saúde, 2014, p.752).

From all the information collected, the deaths were classified either as “fatal work-related accidents” or as “other deaths”. For the first ones, we re-interviewed the family members and coworkers of the victim to obtain a detailed description of the occupational history of the deceased person and field quality control. For this analysis, we classified the fatal work- related accidents found as:

- Work-related accidents by crime (WA/cr): arising primarily from a criminal act, intentional or not, against the worker;
- Strict labor accidents (WA/st): excluded from the previous class, they are those primarily originated from the execution of work activities;
- Work-related accidents in traffic (WA/tr): excluding the previous classes, they are those primarily resulting from collisions, being run over, or falls from motor vehicles or other vehicles in traffic, as well as imbalances or falls of the worker when walking;
- Other work-related accidents (WA/ot): accidents not included in any previous class.
- We started field activities after the study received a favorable opinion from the Research Ethics Committee of the Faculdade de Ciências Médicas of the Universidade Estadual de Campinas (Opinion 918.561).

## RESULTS

We found 415 deaths of residents of Campinas aged between 15 and 65 whose basic causes were external.

When analyzing the autopsy reports performed by the IML for these deaths, we found that 32 were, in fact, not from external causes, but natural deaths. They were excluded from the studied universe, leaving 383 violent deaths to be analyzed. Of them, five (1.3%) were not investigated because we could not identify any relative, friend, or coworker of the deceased person, and we considered them as losses. Thus, the results presented here refer to 378 violent deaths of residents of Campinas aged between 15 and 65 years which happened in or out of the city in 2015.

Table 1 shows the classification of these deaths according to immediate cause.

**Table 1:** Distribution of violent deaths in residents of Campinas, state of São Paulo, Brazil, aged between 15 and 65 years, according to immediate cause, in 2015.

Immediate cause	Absolute frequency (n)	Relative frequency (%)	Work-related accident (absolute and relative)
Murder	160	42.3	25 (15.6)
Traffic accident	117	30.9	42 (35.9)
Suicide	48	12.7	3 (6.3)
Fall	37	9.8	3 (8.1)
Drowning	6	1.6	0 (0)
Other	10	2,6	9 (90.0)
Total	3 78	100	82 (21.7)

In the column 'Work-related accident (absolute and relative)' we present the absolute number of work-related accidents according to the corresponding immediate cause and its proportion in that category, in percentage.

Among the 378 violent deaths found, 82 were classified as work-related accidents, which corresponded to a proportional mortality rate of 21.7%. That is, one in every five violent deaths in this age group among residents of Campinas was caused by a work-related accident.

The magnitude of the incidence of fatal work-related accidents in Campinas is noteworthy. Taking into account the size of the population at risk of work-related accident in the city^4^, in 2015, 10 fatal work-related accidents were identified for every 100,000 inhabitants of economically active age. For males, this coefficient was 17.9 deaths per 100,000 inhabitants in this age group.

The following data refer to the 82 workers identified as victims of work-related accidents. Among the death certificates analyzed, 27 (32.9%) had the “Work-related Accident” field marked as “no”, 50 (61.0%) were marked as “ignored”, and only five (6.1%) were marked as “yes”. Since this is the data source that feeds the Mortality Information System^14^ (SIM) of the Ministry of Health, we can conclude that the underreporting of fatal work-related accidents in the SIM for Campinas in this period would be 93.9% if this information were not corrected from the data of this research.

As for sex, 74 (90.2%) workers were men and eight (9.8%) were women. The mean and median ages at death were 42 and 43 years, respectively. The youngest worker was 18 years old and the oldest was 63 years old. The mean and median educational levels of the group, measured in full years, were seven and eight years, respectively, ranging from zero to 16 years.

Table 2 shows the distribution of the occupations of the 82 deceased workers, according to the Large Groups of the Brazilian Classification of Occupations^15^.

**Table 2:** Classification of occupations of workers killed in 2015, residents of Campinas, state of São Paulo, Brazil, according to the Large Groups of the Brazilian Classification of Occupations.

Large groups	Absolute frequency (n)	Relative frequency (%)
7: Workers in the production of industrial goods and services, except machine operators	28	34.1
5: Service workers, sellers in shops and markets	28	34.1
4: Administrative service workers	7	8.5
1: Senior members of the government, heads of public interest organizations and companies, and managers	5	6.1
2: Professionals of science and arts	4	4.9
6: Agricultural, forestry, hunting, and fishing workers	3	3.7
9: Maintenance and repair workers	3	3.7
0: Military, military firefighters and police	1	1.2
3: Technicians with high school diploma	1	1.2
8: Workers in the production of industrial goods and services, only machine operators	1	1.2
No information	1	1.2
Total	82	100

Regarding labor regulation at the time of death, we found 62 (75.6%) formal workers (15 of them were regulated and self-employed), 12 (14.6%) informal workers, and eight workers (9.8%) of unknown employment relationship.

As to race, 42 (51.2%) were white, 31 (37.8%) were brown, and nine (11.0%) were black. As for religion, 41 (50.0%) were Catholics, 27 (32.9%) were Evangelicals, four (4.9%) were Spiritists, one (1.2%) was a practitioner of *Umbanda* (an Afro-Brazilian religion), five (6.1%) had no religion, and four (4.9%) had unknown religion.

In the evaluation of the use of licit and illicit drugs among deceased workers, 50 (61.0%) used alcohol in the month of death, 28 (34.1%) smoked, 13 (15.9%) used illicit drugs, and 14 (17.1%) had problematic use of alcohol or illicit drugs.

Among the 82 fatal work-related accidents identified, 64 (78.4%) occurred in streets, squares, or highways. Fifty of them (61.0%) occurred outside the working environment.

Table 3 presents the classification of the fatal work-related accidents analyzed.

**Table 3:** Distribution of fatal work-related accidents in 2015, residents in Campinas, state of São Paulo, Brazil, according to type.

Type of work-related accident	Absolute frequency (n)	Relative frequency (%)
Work-related accidents by crime	25	30.5
Strict work-related accidents	19	23.2
Work-related accidents in traffic	35	42.7
Other work-related accidents	3	3.7
Total	82	100

It is surprising the amount of criminal actions we found against workers. Approximately one-third of the fatal work-related accidents in Campinas resulted from homicides.

The Box 1 summarizes the 25 work-related accidents classified as the result of criminal acts (WA/cr).

**Box 1:** Summary description of the twenty-five work accidents directly related to criminal acts that occurred among workers living in Campinas, state of São Paulo, in 2015.

Case	Description
16	Bus driver, as he walked back home from the bus terminal he was beaten and suffocated by two strangers. Motivation: possible disagreement with drug dealers.
24	Cook, he was taken by force by two strangers, who poured a gallon of gasoline on her and set her on fire. Motivation: possible crime of passion
41	Assembler of metallic frames, he was transporting material to a work site in the company car. Stuck in the traffic jam, he was approached by three strangers. One of them fired two shots at the worker. Motivation: possibly confused with a person wanted dead.
42	Rural worker, while pruning guava trees in an orchard, he was approached by a stranger who arrived on a motorcycle and shot three times at the worker. Motivation: possible crime of passion
64	Chief of maintenance at a large hotel, on his way out of work he was attacked by two strangers who beat him to death. Motivation: possibly confused with a person wanted dead.
76	Sex worker (CBO 5198-05), she was stabbed by a client during sex. Motivation: disagreement with client regarding the price of the program.
78	Mechanic, he went to rescue a broken car in a street. While inspecting the engine, he was approached by two strangers who fired two shots at him. Motivation: possible crime of passion
110	Security guard of a gated community, he was working inside a guardhouse and was shot three times in the back, fired from the street by a hooded stranger. Motivation: possible crime of passion
121	A grill man who worked at a snack bar, he was shot several times by strangers as he returned home from work. Motivation: possible disagreement with drug dealers.
123	A recyclable waste picker, she was run over by a bus, maneuvering into a garage, while collecting cardboard in the street. Motivation: accidentally run over while working.
149	Hot dog salesman, shot by strangers while reacting to a robbery. Motivation: robbery.
155	A janitor of a sports club, he was stabbed during work hours by a coworker. Motivation: disagreement with coworker.
159	Woodworker, he was shot several times while reacting to a robbery on the way to work. Motivation: robbery.
174	Pizzaiolo, owner of a small pizzeria, he left work and went to his house during work hours to pick up supplies. He was shot five times when he got out of the car. Motivation: unknown.
187	Building porter, he was stolen, beaten, and killed by three unknown teenagers leaving the building at the end of the shift and heading home. Motivation: robbery.
211	Photocopier operator, he was abducted when leaving work to buy toner, beaten, and abandoned on the side of a highway. Motivation: possible crime of passion
218	Owner of a stationery shop, he was kidnapped from the entrance of the shop by two unknown armed men. His body was found in a thicket days later with beating marks. Motivation: unknown.
219	Bar owner in the periphery, he was hit by several shots fired by a client while working. Motivation: disagreement with customer regarding price of the drink.
220	Correctional officer, after leaving work, he drove his car home when he was approached by two persons in a motorcycle. The passenger fired ten shots at the officer. Motivation: possible disagreement with drug dealers.
247	Masonry worker, he was beaten and killed by his boss's son on his way home from work. Motivation: disagreement with third party.
280	Supermarket assistant, he was hit at the entrance of the work, while unloading goods, by a car that invaded the sidewalk. The car had just been stolen and the driver was being chased by the police at the time of the accident. Motivation: accidentally run over while working.
321	Security guard of a transport company, he was hit during patrol by two rifle shots made by a stranger. Motivation: possibly confused with a person wanted dead.
324	Security guard of a snack bar, he ran into confrontation with two robbers at work and was shot.
359	Bar owner, she was shot by a client. Motivation: disagreement as to the change of the drink.
383	Driver of a microbus, he was killed by a passenger while driving. Motivation: possible disagreement with criminals.

Among the 25 workers who were victims of WA/cr, 19 (76.0%) worked or went from home to work alone, that is, without the company of other coworkers. Of them, 11 (44.0%) were victims of criminal acts that led to death late at night or at dawn, and 10 (40.0%) died in urban areas considered to be of high crime.

The Box 2 briefly describes the 19 work-related accidents classified as strictly related to work activities (WA/st).

**Box 2:** Summary description of the nineteen work accidents strictly related to labor activities that occurred among workers living in Campinas, state of São Paulo, in 2015.

Case	Description
25	Masonry worker, he was laying tiles on a roof, got off balance, and fell to the ground.
95	Highway worker, he was hit while painting a security line at the shoulder of a highway.
100	Panel installer, an billboard fell over him while he was working fixing it.
119	Horse trainer, he suffered a trauma to the thorax from a kick from the horse that he was treating.
126	Truck driver, he fell from the truck body while unloading tiles.
131	Security guard, he suffered a car accident on the highway while escorting a truck.
137	Truck driver, he suffered a car accident on the highway while transporting cargo.
138	Hawker, he was run over at the shoulder of the highway while selling fruit.
160	Construction worker, he was crushed by marble blocks that fell from the body of the truck that he was unloading.
215	Masonry worker, he was laying tiles on a roof, got off balance, and fell to the ground.
221	Recyclable waste picker, he was electrocuted as he tried to move a bare, high-tension wire off a sidewalk from a utility pole while picking up recyclable material.
222	Bus driver, he was helping a wheelchair user enter the vehicle when he slipped and hit his head on the curb.
246	Truck driver, he parked the vehicle on incline, got off, and went behind the truck. At that moment, the brake system failed and the truck came down and pressed the driver against another parked truck.
295	Masonry worker, he drove a tractor on a dirt road on a steep slope, lost control of the vehicle, and flipped over.
303	Electrician, he was electrocuted and fell from the top of a pole while making a clandestine connection.
314	A parcel dispatcher, he crashed his motorcycle into a car while he was bringing parts to a parking lot.
340	Masonry worker, he was buried by a high wall that collapsed on him while working on a construction site.
403	Masonry worker, he was hit by a rafter that fell from the eighth floor of a building under construction while working on a construction site.
411	Truck driver, he transported goods, stopped at the shoulder to answer the phone, got out of the truck, and was hit by a car.

The 35 (42.7%) work-related accidents classified as WA/tr were from collisions and being run over on urban roads and highways. The three (3.7%) work-related accidents classified as WA/ot were suicides, all committed in the work environment during work hours. In one, a handyman, chronically exposed to organophosphates, hanged himself while trimming grass in a public garden. In another one, an industrial painter, chronically exposed to organic solvents, deliberately threw himself at a bus that passed in front of the industrial gate at the exit of the work. The third worker was the owner of a small wood shop who hanged himself in his office during office hours, leaving a letter stating that he could no longer withstand the pressure of creditors at his establishment demanding late payments.

## DISCUSSION

The amount of work-related accidents found is alarming by itself, mainly because it refers to absolutely preventable events, expressing negligence and social injustice^19^.

In this study, we avoided to use the classic categorization of work-related accidents as “typical” and “commute”. We did so because these terms lead, at least in the common sense, to an outdated and even misconceived conception. Work-related accidents cannot be labeled as typical. Typical is what normally happens, which is characteristic, which serves as a model. The Brazilian Ministry of Social Security calls as typical the accidents “ from the characteristic of the professional activity performed by the injured person”^12^, a definition that naturalizes the work-related accident, limits its recognition, and attributes it to situations more or less expected and restricted to the performance of work activities. We also did not use the category “commute” because this term does not specify the accident. When commuting, the worker suffers not only accidents limited to locomotion, but also accidents strictly from the work activity, as well as intentionally criminal actions. In addition, the use of the “commute” category frequently leads to endless discussions of strictly pecuniary interest on the nature of the path, path habituality, path changes, intentional path interruptions, etc.

The study on work and its repercussions on the life of the worker should suggest not only the analysis of issues directly related to work but also its articulation with the whole society of which it is a product and with which it interacts^11^. The classification of work-related accidents we used here categorized fatal accidents as WA/cr, WA/st, WA/tr, and WA/ot for two purposes. On the one hand, we wanted to highlight that the organization of work and the ways in which the worker develops his or her work activities are only an important part of the problem. On the other hand, we wanted to give greater visibility to the reflexes of the changes in the labor market in recent decades (with the expansion of the service sector and the greater exposure of workers to the street environment) in the current context of Brazilian social violence, expressed by crime, aggressiveness of traffic, and the increase in the incidence of suicide^8^.

Among all the fatal work-related accidents found, 30.5% were classified as WA/cr. The WA/ cr results from criminal acts practiced intentionally or not by coworkers, clients, the general public, criminals, police officers, etc. In addition to the enormous underreporting of fatal work-related accidents resulted from the removal of most of the working population from the official WA notification system, as well as the underreporting of fatal work-related accidents resulted from failures to notify the system, the WA/cr is also generally not recognized as work- related accident, and it is classified in official statistics as common violence (a term that in itself denounces the seriousness and banalization of violence in our country), contributing to the invisibility of adverse work situations responsible for its occurrence^7^.

Souza et al. (2006) have report that “work-related homicides would be conditioned by risk factors specific to some activities: solitary or small group work, exchange of money with the public, work performed late at night or early morning, areas of high crime, custody of valuable goods, and work on the streets”^22^ (p.87). This view is compatible with the findings presented here. As seen, of the 25 WA/cr identified, 76.0% occurred in situations where the worker was alone in the work or in the street, 44.0% occurred late at night or at dawn, and 40.0% occurred in areas of high crime. Social violence in Brazil is a complex phenomenon, rooted in the social inequalities that the country has created throughout its development. Control of violence and its impact on the world of work is also a complex and macrostructural issue. Under an emergency and palliative character, we can identify that the situations listed above are risks that, when possible, should be avoided in order to reduce the occurrence of WA/cr.

Regarding the 19 WA/st identified, more than half occurred in situations such as falls from height, heavy objects falling on the person, electrocution, and being buried, that is, events for which the current legislation already prescribes the adoption of prevention barriers, monitoring, and mitigation of effects. It is up to the Occupational Health Reference Centers (CEREST) and the representation of workers to act politically to ensure compliance with the law.

In addition, among the fatal work-related accidents analyzed, 42.7% were classified as WA/tr. After excluding the WA/cr and WA/st, these 42.7% refer only to accidents in traffic, corresponding to being run over, collisions between cars or motorcycles, and falls from vehicles in motion. In addition to the reflexes of the saturation of cars in big cities and the Brazilian transport policy that favors individual transport over the collective, we cannot fail to recognize that the WA/tr also has as one of the components of its genesis, under an epidemiological view, the physical and mental stress to which the workers are submitted. In addition, most of these accidents are not recognized in official statistics as work-related accidents. We found that the most of the families of WA/tr victims were concerned with the procedures necessary to obtain the Brazilian road insurance^23^, which is widely publicized in the media. On the other hand, the recognition of WA/tr as related to work and initiatives to obtain the accidental death pension^12^ by the families of the deceased workers were minimal.

The WA/cr, WA/tr, and WA/ot were responsible for three-fourths of the fatal work-related accidents analyzed here. In fact, 78.4% of the fatal work-related accidents identified occurred in streets, squares, and highways. We also observed that 61.0% of the fatal work-related accidents occurred outside the working environment of the victims. This reinforces the need to rethink strategies for the prevention of work-related accidents and safeguarding of the health of Brazilian workers. Preventive actions in the area of Occupational Health need to go beyond the limits of the “factory space” and act on this reality.

We also need to rethink the information system in order to improve the identification of this type of event, contributing with the construction of effective interventions to mitigate risks and prevent absolutely preventable work-related accidents. To this end, it helps to understand the work-related accident as any acute event, either intentional or fortuitous, that causes the death of the worker or affects his or her physical or mental integrity, which occurs during the exercise of the work or in the movements needed for it. This conception is different from the official one, advocated by the Ministry of Social Security (Law 8213, 1991). From this point of view, the fundamental information to be obtained is: what was the activity of the individual at the time of the accident that has led to his or her death? This conception of work-related accident and the concern to investigate this question are not usually contemplated by the teams that feed the SIM in the municipalities. The discussion and update of the concept of work accident in society as a whole and in particular among health professionals and students would contribute to reduce the invisibility of work- related accidents. The relationship between social violence and work-related accidents also needs to be discussed. In addition, protocols should be updated and data collection teams should be trained as widely as possible in Brazilian municipalities, under this understanding. The latter is a costly, difficult, long-term investment that will only yield results if it complements the previous proposal.

It is interesting to note that in this study, among the deceased persons, formal workers (75.6%) prevailed in relation to informal workers. In the study carried out by Hennington et al.^3^, with data collected 15 years ago with a similar method also in Campinas, only 30% of the deceased persons were formal workers. In the study of Lacerda et al.^6^, whose data were collected 11 years ago in Salvador, only one-third of the deceased workers were classified as formal. Perhaps this discrepancy is partly due to the increase in the proportion of formal jobs that took place in Campinas in the last decade. According to the 2014 Annual Report of Social Information (RAIS), the level of formal employment increased by 1.27% compared to 2013, with an increase of 623,077 jobs, confirming the growth trajectory of formal employment in the country between 2010 and 2015. According to *Fundação Seade* ^2^, the administrative region of Campinas represents 15.9% of the formal jobs of the state. It is estimated that, in July 2015, the proportion of formal workers in the Metropolitan Region of São Paulo, less than 100 km from Campinas, was 57% of the employed population^5^, a figure that can be extrapolated to Campinas.

We highlight that the information losses of this study were due to the non-identification of informants of only five (1.3%) deaths from the total of 383 violent deaths that occurred in Campinas in 2015. Some factors contributed to this. Most of the interviews were carried out approximately one to two weeks after the deaths, except for those who died outside Campinas. This minimized the occurrence of problems resulting from changes of addresses of relatives after death. Moreover, when there was a change of address, we could interview neighbors and coworkers. The use of information on violent deaths obtained in the written and spoken press was also important, not only for the identification of informants, but mainly for the enrichment of the stories. Despite the caution with the sensationalist behavior of many means of communication of this type of news, which often overestimate aspects under research, some authors have indicated that newspapers are an important complementary source of qualification and complementation of information on deaths from external causes^24^, specifically regarding fatal work-related accidents^22^.

## FINAL CONSIDERATIONS

There are more than 1,100,000 persons in Campinas. The city is home to one of the Metropolitan Regions of Brazil. The situation described here does not appear to be a particular case. On the contrary, this appears to be the condition found, to a greater or lesser extent, throughout the country. Along with old problems, the deterioration of the Brazilian political, social, economic, and environmental scenario causes great transformation in social and work relations. The increasing deregulation and precariousness of work, the increase in unemployment, and the presence of organized groups mediating conflicts outside the Government in the peripheries of great urban centers have imposed a significant change in the mortality profile of Brazilian workers. Today, any preventive action in the area of Occupational Health must contemplate this reality. In doing so, we can dream of implementing more effective measures to prevent and control work-related accidents, beginning to recover a debt with millions of Brazilian workers who struggle daily for survival, who contribute with the development of Brazil, whose accidents are not even computed, and whose families cannot enjoy protection and solidarity.
